# Supplementary figures and images for: Human-wildlife conflicts with crocodilians, cetaceans and otters in the tropics and subtropics
Source: PeerJ. 2022 Jan 4;10:e12688. doi: 10.7717/peerj.12688 (PMC8740516; doi:10.7717/peerj.12688)

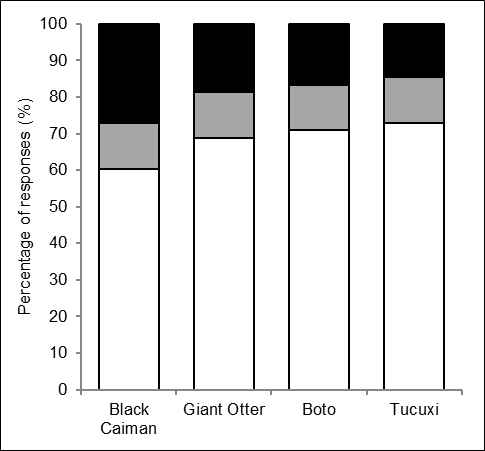

Supplement: Supplemental Information 3 — Percentage of interviewees who believe that coexistence with black caiman, giant otters, botos and tucuxi is possible (white), not possible (black), or depends on various factors (grey). [file peerj-10-12688-s003.png]
