# Supplementary material for: Human-wildlife conflicts with crocodilians, cetaceans and otters in the tropics and subtropics
Source: PeerJ. 2022 Jan 4;10:e12688. doi: 10.7717/peerj.12688 (PMC8740516; doi:10.7717/peerj.12688)
Supplement: Supplemental Information 4 — The two covariates used were the fluvial distance from Carauari and percentage of várzea in a 5 km buffer around the community. [file peerj-10-12688-s004.docx]

Table S1. Results for binary logistic regression in response to interview questions (n= 49) in the Médio Juruá case study. The two covariates used were the fluvial distance from Carauari and percentage of *várzea* in a 5 km buffer around the community.

| **Covariate** | **Fluvial distance from Carauari** | | | **Percentage *várzea* in 5 km buffer** | | |
| --- | --- | --- | --- | --- | --- | --- |
| Species and Question | B | Exp(B) | P value | B | Exp(B) | P value |
| **Black Caiman**  Causes Problems  Damages Equipment  Entangled in Net  Frighten Fish  Leave Areas to Fish  Hunted in Community  Hunted in 2013/2014  Hunted in Life | -  -  0.003  -0.002  -0.001  0.003  -0.003  0.001 | -  -  1.003  0.998  0.999  1.003  0.997  1.001 | -  -  0.499  0.527  0.804  0.291  0.317  0.661 | -  -  -0.018  -0.009  0.002  0.011  -0.004  0.013 | -  -  0.982  0.992  1.002  1.011  0.996  1.013 | -  -  0.317  0.546  0.931  0.455  0.803  0.379 |
| **Giant Otter**  Causes Problems  Damages Equipment  Entangled in Net  Frighten Fish  Leave Areas to Fish  Hunted in Community  Hunted in 2013/2014  Hunted in Life | 0.000  0.007  -0.004  0.013  0.003  -0.009  -0.008  0.000 | 1.000  1.008  0.996  1.013  1.003  0.991  0.992  1.000 | 0.947  **0.009**  0.203  0.479  0.189  0.116  0.305  0.935 | 0.039  0.030  0.009  0.009  0.008  0.007  0.021  0.023 | 1.040  1.030  1.009  1.009  1.008  1.007  1.021  1.023 | 0.252  0.061  0.552  0.865  0.584  0.695  0.382  0.152 |
| **Boto**  Causes Problems  Damages Equipment  Entangled in Net  Frighten Fish  Leave Areas in Fish  Hunted in Community  Hunted in 2013/2014  Hunted in Life | -  -  0.004  0.006  -0.002  -0.003  -0.010  0.003 | -  -  1.004  1.006  0.998  0.997  1.010  1.003 | -  -  0.252  0.081  0.609  0.570  0.510  0.475 | -  -  0.001  0.007  0.018  0.006  -48.983  0.010 | -  -  1.001  1.007  1.018  1.006  0.000  1.010 | -  -  0.934  0.659  0.318  0.806  0.942  0.699 |
| **Tucuxi**  Causes Problems  Damages Equipment  Entangled in Net  Frighten Fish  Leave Areas to Fish  Hunted in Community  Hunted in 2013/2014  Hunted in Life | -0.010  -0.010  0.005  -0.007  -  -0.009  -  - | 0.990  0.990  1.005  0.993  -  0.991  -  - | 0.350  0.350  0.080  0.110  -  0.521  -  - | 0.025  0.025  0.025  -0.012  -  0.023  -  - | 1.025  1.025  1.025  0.988  -  1.024  -  - | 0.363  0.363  0.095  0.549  -  0.533  -  - |
